# Alexithymia and dissociation in personality disorders: a retrospective cross-sectional study

**DOI:** 10.1192/j.eurpsy.2022.1709

**Published:** 2022-09-01

**Authors:** M. Pacetti, D. Salmaso

**Affiliations:** 1 DSM-DP , Centro Salute Mentale Forlì, Italy, Forlì, Italy; 2 DSM-DP , Centro Salute Mentale Forlì, Italy, Forli, Italy

**Keywords:** Alexithymia, dissociation, personality disorders

## Abstract

**Introduction:**

Patients with “personality disorder”, has history of traumatic life events and are predisposed to develop alexithymia and dissociation, considered as risk factor for severity.

**Objectives:**

The aim of the research is to analyze alexithymia relating to dissociative symptoms, and investigate their associations, in 34 patients with personality disorder.

**Methods:**

Outpatients with personality disorder relating to Mental Health Centre have been identified and tested with the Dissociative Experiences Scale, the Parma Scale for Personality Functioning and the Toronto Alexithymia Scale.

**Results:**

There was no significant association between age of patients and presence of alexithymia (r=-0.16) and dissociation (r=-0.19); most patients with alexithymia and dissociation were female (67%; 0.67%). 71% of alexithymic subjects had attended lower secondary school, 50% upper secondary school and 43% had a university degree. Substance use is higher in alexithymic patients (73%). 69% of subjects who do not undergo any individual or group psychotherapy are alexithymic; for dissociative symptoms it is significant to undergo both psychotherapies. Alexithymia and dissociation are more frequent in histrionic personality disorder (80%; 60%) and borderline personality disorder (55%; 54%). There is a potential correlation between alexithymia and the presence of dissociative symptoms (r=0.64).

**Conclusions:**

This study found that alexithymia and dissociative symptoms are frequent within personality disorders, particularly in histrionic and borderline personality disorder. We found that the two phenomena were associated. Furthermore we found alexithymia is more influenced by external factors than dissociative symptoms.

**Disclosure:**

No significant relationships.

